# A Simple Allergist-Led Intervention Improves Resident Training in Anaphylaxis

**DOI:** 10.1155/2016/9040319

**Published:** 2016-02-21

**Authors:** Artemio M. Jongco, Sheila Bina, Robert J. Sporter, Marie A. Cavuoto Petrizzo, Blanka Kaplan, Myriam Kline, Susan J. Schuval

**Affiliations:** ^1^Division of Allergy & Immunology, Hofstra Northwell School of Medicine, 865 Northern Boulevard, Suite 101, Great Neck, NY 11021, USA; ^2^Center for Immunology and Inflammation, Feinstein Institute for Medical Research, 350 Community Drive, Manhasset, NY 11030, USA; ^3^Pediatric Allergy & Immunology, Stony Brook Children's Hospital, T-11, Room 080, Stony Brook, NY 11794, USA; ^4^Division of Allergy & Immunology, University of South Florida, 140 7th Avenue South, CRI 4008, St. Petersburg, FL 33701, USA; ^5^ENT & Allergy Associates, 261 Fifth Avenue, Suite 901, New York, NY 10016, USA; ^6^ProHealth Care LLP, 2 Lincoln Avenue, No. 302, Rockville Centre, NY 11570, USA; ^7^Biostatistics Unit, Feinstein Institute for Medical Research, 350 Community Drive, Manhasset, NY 11030, USA

## Abstract

Physicians underrecognize and undertreat anaphylaxis. Effective interventions are needed to improve physician knowledge and competency regarding evidence-based anaphylaxis diagnosis and management (ADAM). We designed and evaluated an educational program to improve ADAM in pediatrics, internal medicine, and emergency medicine residents from two academic medical centers. Anonymous questionnaires queried participants' demographics, prior ADAM clinical experience, competency, and comfort. A pretest assessing baseline knowledge preceded a 45-minute allergist-led evidence-based presentation, including practice with epinephrine autoinjectors, immediately followed by a posttest. A follow-up test assessed long-term knowledge retention twelve weeks later. 159 residents participated in the pretest, 152 participated in the posttest, and 86 participated in the follow-up test. There were no significant differences by specialty or site. With a possible score of 10, the mean pretest score (7.31 ± 1.50) was lower than the posttest score (8.79 ± 1.29) and follow-up score (8.17 ± 1.72) (*P* < 0.001 for both). Although participants' perceived confidence in diagnosing or managing anaphylaxis improved from baseline to follow-up (*P* < 0.001 for both), participants' self-reported clinical experience with ADAM or autoinjector use was unchanged. Allergist-led face-to-face educational intervention improves residents' short-term knowledge and perceived confidence in ADAM. Limited clinical experience or reinforcement contributes to the observed decreased knowledge.

## 1. Introduction

Teaching physicians effectively about low probability, high consequence medical conditions, such as anaphylaxis, is challenging. Medical education curricula emphasize more common high stakes conditions (e.g., stroke) where misdiagnosis or mismanagement leads to poor outcomes. Physicians may lack opportunities to gain firsthand clinical experience or to reinforce their limited learning of infrequent conditions. Effective interventions are needed.

Clinicians face several challenges when dealing with anaphylaxis, a potentially life-threatening allergic reaction requiring immediate identification and treatment. First, there are no universally accepted diagnostic criteria for anaphylaxis [1, 2]. A comprehensive clinical definition of anaphylaxis from an NIH expert panel has not achieved widespread acceptance among physicians despite high reported sensitivity and negative predictive value [1, 3, 4]. Second, there are no pathognomonic anaphylaxis signs or symptoms. Physicians may overlook the diagnosis because the clinical presentation may vary among patients, even in the same patient with a history of multiple episodes [1, 5]. Third, physicians may not consider anaphylaxis when patients do not present stereotypically (e.g., laryngeal edema after bee sting). Additionally, diagnostic tests are rarely useful during an acute episode. Furthermore, the usual probability-based methods of clinical reasoning and decision-making are difficult to apply to anaphylaxis [6, 7]. Estimates of anaphylaxis prevalence and incidence are unclear due to the lack of symptom recognition, poor physician awareness of diagnostic criteria [1, 2], and paucity of robust, validated methods for identifying anaphylaxis diagnoses using currently available administrative claims [8, 9]. Thus, it is unsurprising that medical personnel underrecognize and undertreat anaphylaxis [1, 2].

Anaphylaxis diagnosis and management (ADAM) by physicians need improvement, regardless of the stage of training [10–22]. Despite the establishment and dissemination of treatment guidelines, medical providers consistently underutilize or incorrectly administer and dose epinephrine, which is accepted as first-line treatment [10–27]. Instead of prompt epinephrine administration, practitioners continue to utilize second-line agents, such as antihistamines and glucocorticoids, contrary to evidence-based recommendations [10–29].

Improving provider education regarding ADAM is an unmet but modifiable deficiency [30–33]. Allergists are particularly well suited to address this gap, as evidenced by the few published studies reporting successful allergist-led interventions [34–36]. We developed, implemented, and evaluated an educational program consisting of face-to-face didactic session and hands-on training conducted by allergy trainees or attending physicians in the proper use of epinephrine autoinjectors. We hypothesized that this allergist-led intervention would improve residents' knowledge, competence, and perceived confidence in anaphylaxis diagnosis and management. Allergists and likely other nonallergist providers can adapt our simple and resource nonintensive intervention in a variety of settings to educate other providers about evidence-based ADAM.

## 2. Methods

### 2.1. Study Description and Eligibility

This longitudinal study examined full-time resident physicians at all training levels who were enrolled in an Accreditation Council for Graduate Medical Education, accredited training program in internal medicine (*N* = 204), pediatrics (*N* = 153), or emergency medicine (*N* = 40), from July 2010 to June 2013, at tertiary care university hospitals in two health systems. Residents were recruited during an hour-long educational conference in which they received an explanatory letter about the study and asked to participate. To maximize participation and account for differences in academic schedules, residents were recruited during two distinct department-specific conferences on different academic blocks. The institutional review boards at both institutions approved this study and waived the need to obtain written informed consent from participants.

### 2.2. Intervention, Quizzes, and Questionnaire

At the recruitment session, residents completed an anonymous questionnaire which queried participants' demographics, prior clinical experience, perceived competency, and comfort with ADAM, as well as a 10-item multiple choice pretest that assessed baseline knowledge of anaphylaxis. Attending physicians (Artemio M. Jongco and Susan J. Schuval) and/or trainees (Sheila Bina and Robert J. Sporter) from the Division of Allergy and Immunology from the respective institutions presented a 45-minute evidence-based didactic lecture using PowerPoint (Microsoft, Redman, WA), followed by hands-on practice with needleless epinephrine autoinjector trainers (EpiPen® trainer). Study personnel observed participants' technique, provided constructive criticism when appropriate, and answered participants' questions related to the educational content or autoinjector use. Immediately following the presentation, the residents completed a similar 10-item posttest to evaluate knowledge acquisition. Approximately 12 weeks later, during another nonanaphylaxis conference, residents completed a similar 10-item follow-up quiz and questionnaire. To foster a safe and nonpunitive learning environment, only residents and study personnel were present at conference sessions. No identifying information was collected from the participants, nor were identifiers recorded on quizzes or questionnaires. Hence, linking individual's responses at different time points or connecting an individual's performance to his/her identity was not possible. Only residents who participated in both programs were included in the analysis. Examples of the questionnaires and quizzes are provided in Supplementary Material available online at http://dx.doi.org/10.1155/2016/9040319.

The authors developed the quizzes, consisting of clinical scenarios that evaluated knowledge of evidence-based anaphylaxis diagnosis and management. To ensure that quiz questions were roughly equivalent in complexity, the scientific content was identical from one quiz to another, with minor modifications (e.g., clinical parameters, order of questions, and answer choices). Quizzes were graded numerically on a scale from 0 to 10. Before the study, the authors reviewed the scientific content of the educational intervention, quizzes, and answers. The authors pilot-tested the quiz questions on a small group of rotating residents in the Division of Allergy & Immunology at Hofstra Northwell School of Medicine. Participants did not have access to quiz answers at any point during the study. Achieving 8 correct answers on the quiz was considered to be the minimum level of competence for medical knowledge.

### 2.3. Statistical Analysis

All statistical analyses were conducted using SAS 9.3 (SAS Institute Inc., Cary, NC). Graphs were generated using Prism 6 (GraphPad Software, San Diego, CA). The results of the descriptive analysis were shown as mean ± standard deviation with 95% confidence intervals and as percentages. The chi-square test was used to measure the association between the categorical variables, and Wilcoxon rank sum test or Kruskal-Wallis test was used to compare the groups on the continuous variables. A two-tailed *P* < 0.05 was considered significant. Bonferroni adjustment was applied for multiple comparisons.

## 3. Results

The two academic health centers employed a total of 397 residents that were eligible to participate (204 internal medicine, 153 pediatrics, and 40 emergency medicine residents). A total of 159 residents participated in the pretest (response rate of 40.05%). One hundred and fifty-two residents of the original 159 (95.60%) completed the posttest, and 86 residents (54.09%) were available for the follow-up test.  Table 1 provides the distribution of sample characteristics by site and specialty. Since chi-squared analysis failed to reveal a significant difference by site (*P* = 0.86) or by specialty (*P* = 0.95) over time, data were combined in subsequent analyses.

Table 2 summarizes participant characteristics at baseline and follow-up. Chi-squared analysis revealed that the proportion of participants having demonstrated epinephrine autoinjector use increased from baseline to follow-up (*P* = 0.006). The proportion of participants who had diagnosed (*P* = 0.06) or managed (*P* = 0.06) anaphylaxis or used an epinephrine autoinjector (*P* = 0.08) in the past did not differ significantly from baseline to follow-up. Of the participants who reported having managed anaphylaxis prior to the study, the most common venue was the emergency department (42.35%), followed by general ward (20.59%), intensive care unit (24.71%), and then allergy office (2.35%) (data not shown). Also, the proportion of participants who self-reported being confident in their ability to diagnose (*P* < 0.001) or manage (*P* < 0.001) anaphylaxis increased from baseline to follow-up.  Table 3 summarizes participants' self-reported behaviors and attitudes at follow-up. During the interval since the intervention, despite increased self-reported confidence in ADAM, residents appear to have had few opportunities to utilize what they have learned about anaphylaxis, or to refer patients with anaphylaxis to allergists.

**Figure 1:**
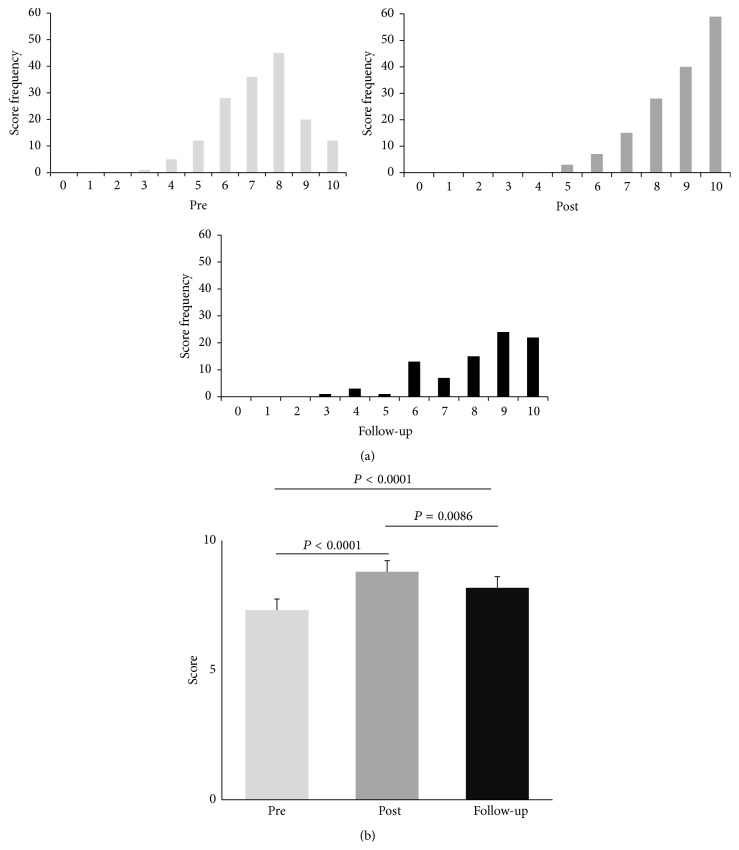
Summary of quiz scores. (a) Distribution of quiz scores at different time points. (b) Mean quiz scores at different time points. Error bars represent standard deviation.

Figure 1(a) illustrates the nonnormal distribution of quiz scores. The mean pretest score of 7.31 ± 1.50 (95% CI 7.08–7.54) was lower than the posttest score of 8.79 ± 1.29 (95% CI 8.58–9.00) and follow-up score of 8.17 ± 1.72 (95% CI 7.80–8.54).  Figure 1(b) shows that the distribution of pretest scores is significantly lower than posttest scores and follow-up scores (*P* < 0.001 for both). The distribution of follow-up scores is significantly lower than that of posttest scores (*P* = 0.0086). These results were significant even after Bonferroni adjustment was made for multiple comparisons (*α* = 0.017). Furthermore, our intervention appears to have helped the participants achieve the medical knowledge competency threshold of 8 correct answers.

Table 4 lists possible covariates of quiz score. Quiz scores at the three time points did not significantly differ by specialty (*P* = 0.59) or by training level (*P* = 0.62). Moreover, the quiz scores did not vary according to their self-reported experience of past anaphylaxis diagnosis (*P* = 0.10), management (*P* = 0.09), past use (*P* = 0.49), demonstration of epinephrine autoinjector (*P* = 0.16), or past referral to allergist (*P* = 0.37). However, quiz scores did significantly differ depending on residents' reported confidence in the ability to diagnose (*P* = 0.01) or manage (*P* = 0.004) anaphylaxis.

## 4. Discussion

In this study, we demonstrate that allergist-led didactic lectures and hands-on practice with epinephrine autoinjectors are effective educational interventions that enhance short-term resident knowledge of evidence-based ADAM. These findings corroborate the literature which shows that the continuing opportunities to apply knowledge and to practice skills are essential to maintain knowledge and competency [34, 35]. Indeed, the majority of participants reported having limited opportunity to apply or utilize their new knowledge or skills in the 12-week interval between intervention and follow-up. We suspect that resident performance would have continued to decline in the absence of educational reinforcement if reevaluated after 12 weeks.

There are several findings, which failed to reach statistical significance, that further underscore the importance of continuing medical education and ongoing opportunities to practice clinical skills in order to maintain ADAM proficiency. There were trends to suggest an increase in the proportion of participants who had diagnosed (*P* = 0.06) or managed (*P* = 0.06) anaphylaxis or used an epinephrine autoinjector (*P* = 0.08) at follow-up compared to baseline. Moreover, past anaphylaxis diagnosis (*P* = 0.10) and management (*P* = 0.09) are likely covariates of quiz score. Further research is needed to identify the optimal frequency and modality of continuing medical education that will result in maximal retention of knowledge and competency.

Interestingly, study participants reported high levels of confidence in diagnosing or managing anaphylaxis at baseline and follow-up, despite limited clinical experience. In fact, the levels of self-reported confidence increased from baseline to follow-up. This observed disconnect between physician self-assessment and objective measures of competence is unsurprising since physicians have a limited ability for self-assessment [37]. Physicians' overestimation of their own competence may compromise patient safety and clinical outcomes. It may be beneficial to help physicians at all training levels to become more cognizant of this disconnect. Moreover, training programs should consider restructuring current educational endeavors to include increased allocation of time and resources for educating trainees about low probability, high consequence conditions like anaphylaxis, since simple, non-resource-intensive interventions, such as the one described in this paper, can lead to measurable improvements in resident knowledge, and possibly clinical competence.

We acknowledge that our medical knowledge competency threshold score of 8 is somewhat arbitrary and may not necessarily reflect clinical competence. Clinical competence, which relies upon a foundation of basic clinical skills, scientific knowledge, and moral development, includes a cognitive function (i.e., acquiring and using knowledge to solve problems); an integrative function (i.e., using biomedical and psychosocial data in clinical reasoning); a relational function (i.e., communicating effectively with patients and colleagues); and an affective/moral function (i.e., the willingness, patience, and emotional awareness to use these skills judiciously and humanely) [38]. Although evaluating medical knowledge through a quiz represents an incomplete assessment of clinical competence at best, it is still reasonable to hypothesize that medical knowledge correlates with clinical competence to some degree and that lower levels of medical knowledge may negatively impact the quality and efficacy of care delivered by the provider. Thus, the observation that the follow-up quiz scores trended back down towards baseline is worrisome for a possible concomitant decline in the quality of care delivered by the residents. Whether the residents demonstrated any change in their clinical practice after the intervention is unknown and outside the scope of the current study. More research is needed to determine the effect of educational interventions such as this in real-life clinical practice.

This study has several limitations. First, only 40% of all eligible participants were included in the study, and only about half of these participated in the 12-week follow-up evaluation. This is likely due to scheduling difficulties and competing demands on resident time, although we cannot exclude the possibility of participation bias. Notably, our participation rate is similar to other studies of physicians and residents [39–41].

Second, since we did not collect identifying information, we could not ensure that recruited participants completed the entire study, track individual performance over time, or give participants personalized feedback on their quiz performance.

Third, extensively validated quiz questions (e.g., questions from previous certification examinations) were not used due to the lack of access. The content of each question on the quizzes was directly linked to each one of our evidence-based learning goals, thus serving as a measure of face validity. Further, there was consensus among the board certified allergists/content experts who developed, verified, and honed the quiz questions, thereby providing us with a measure of content validity. Finally, because the quizzes were utilized at more than one site, in more than one clinical department, and on a modest sample size, we believe that the generalizability of the instrument was attained to a respectable degree.

Finally, this study only utilized traditional educational modalities of didactic lecture and hands-on practice. More research is needed to evaluate the efficacy of various educational interventions, especially with regard to long-term knowledge retention, improved performance on objective measures of clinical competence, and actual patient outcomes. Simulation-based education may hold promise in this regard [30–32, 35].

This study had several strengths. It was multicenter and included participants from multiple specialties. Also, the larger sample size of this study and the longer follow-up interval distinguish this study from other published studies of educational interventions. Furthermore, since the intervention is relatively simple and not resource-intensive, it can be adapted and implemented in a variety of educational settings.

## 5. Conclusion

Physicians, regardless of the stage of training, underdiagnose and undertreat anaphylaxis. Teaching providers about evidence-based ADAM is challenging. The allergist-led face-to-face educational intervention described above improves residents' short-term knowledge, competence, and perceived confidence in ADAM. Lack of clinical experience and/or educational reinforcement may contribute to knowledge and competence decline over time. Thus, continuing medical education, coupled with ongoing opportunities to apply knowledge and practice skills, is necessary. Innovative educational interventions are needed to improve and maintain resident knowledge and clinical competence regarding evidence-based ADAM. More research is also needed to determine the impact of such interventions on patient outcomes.

## Supplementary Material

The educational materials generated for this study are included as Supplementary Material. Appendix 1 contains the slides presented by the authors during the 45 minute evidence-based didactic lecture. Appendix 2 and 3 are sample quizzes developed by the authors. Appendix 2 is a pre-test and Appendix 3 is a post-test/follow-up test. Appendix 4 and 5 are sample questionnaires developed by the authors to query demographics and participant experience with anaphylaxis diagnosis and management. Appendix 4 is a pre-test questionnaire, and Appendix 5 is a post-test/follow-up questionnaire.

## Figures and Tables

**Table 1:** Sample characteristics by site and specialty.

	Pretest	Posttest	Follow-up test	Total	*P* value
	*N* (%)	*N* (%)	*N* (%)		
Location					
Health system 1	88 (55.4)	80 (52.6)	45 (52.3)	213	0.86
Health system 2	71 (44.6)	72 (47.4)	41 (47.7)	184	
Specialty					
Pediatrics	60 (37.7)	58 (38.2)	35 (40.7)	153	0.95
Internal medicine	84 (52.8)	79 (52.0)	41 (47.7)	204	
Emergency medicine	15 (9.5)	15 (9.8)	10 (11.6)	40	

**Table 2:** Summary of sample demographic characteristics.

Characteristic	Pretest *N* (%)	Follow-up test *N* (%)	*P* value
Gender			
Male	64 (43.2)	38 (45.8)	0.71
Class year			
PGY1	77 (52.0)	48 (55.8)	0.81
PGY2	34 (23.0)	17 (19.8)	
PGY3	37 (25.0)	21 (24.4)	
US medical school graduate			
Yes	109 (73.6)	63 (73.3)	0.95
Diagnosed anaphylaxis in past			
Yes	49 (33.1)	39 (45.4)	0.06
Managed anaphylaxis in past			
Yes	74 (50.0)	54 (62.8)	0.06
Used epinephrine autoinjector in past			
Yes	13 (8.8)	14 (16.3)	0.08
Demonstrated epinephrine autoinjector in past			
Yes	55 (37.2)	48 (55.8)	0.006
Referred patient to allergist in past			
Yes	53 (35.8)	30 (34.8)	0.89
Confidence in diagnosing anaphylaxis			
Yes	90 (60.8)	72 (83.7)	<0.001
Confidence in managing anaphylaxis			
Yes	77 (52.4)	68 (79.1)	<0.0001

**Table 3:** Summary of self-reported behaviors and attitudes at 12-week follow-up.

Behavior and/or attitude	*N* (%)
Diagnosed anaphylaxis since lecture	21/86 (24.42)
Managed anaphylaxis since lecture	25/86 (29.07)
Used epinephrine autoinjector since lecture	9/86 (10.47)
Demonstrated epinephrine autoinjector since lecture	16/86 (18.60)
Referred patient to allergist since lecture	19/86 (22.09)
Confidence in diagnosing anaphylaxis since lecture	77/86 (89.53)
Confidence in managing anaphylaxis since lecture	79/86 (91.86)

**Table 4:** Possible covariates of quiz scores.

Variable	*P* value
Specialty	0.59
Level of training	0.62
Diagnosed anaphylaxis in past	0.10
Managed anaphylaxis in past	0.09
Used epinephrine autoinjector in past	0.49
Demonstrated epinephrine autoinjector in past	0.16
Referred patient to allergist in past	0.37
Confidence in diagnosing anaphylaxis	0.01
Confidence in managing anaphylaxis	0.004
