# Supplementary material for: A Simple Allergist-Led Intervention Improves Resident Training in Anaphylaxis
Source: J Allergy (Cairo). 2016 Feb 21;2016:9040319. doi: 10.1155/2016/9040319 (PMC4779520; doi:10.1155/2016/9040319)
Supplement: Supplementary file 1 — The educational materials generated for this study are included as Supplementary Material. Appendix 1 contains the slides presented by the authors during the 45 minute evidence-based didactic lecture. Appendix 2 and 3 are sample quizzes developed by the authors. Appendix 2 is a pre-test and Appendix 3 is a post-test/follow-up test. Appendix 4 and 5 are sample questionnaires developed by the authors to query demographics and participant experience with anaphylaxis diagnosis and management. Appendix 4 is a pre-test questionnaire, and Appendix 5 is a post-test/follow-up questionnaire. [file 9040319.f1.pdf]

Appendix 1: Presentation

Anaphylaxis for House Staff:
Rapid Recognition and Treatment

2:00 am – Rapid Response

You are covering a patient with urosepsis.

She is receiving her second dose of ceftriaxone.

You are called for **wheeze**, **skin rash**, and **hypotension**.

Are you prepared to handle this situation?

Recognizing Anaphylaxis

- Anaphylaxis can be DIFFICULT to recognize
  - Varied symptoms
  - Variable severity
- Anaphylaxis is a SERIOUS ALLERGIC REACTION
  - RAPID ONSET after exposure to an allergen
  - MAY BE FATAL
  - Ideally recognized before progression to life-threatening symptoms, including SHOCK

Diagnostic Criteria

- Developed by a multi-disciplinary group of experts*
- To help clinicians recognize the full spectrum of signs and symptoms of anaphylaxis
- 3 CRITERIA:
  - Each represents a DIFFERENT PRESENTATION of anaphylaxis
  - Meeting any ONE CRITERIA = anaphylaxis

*Sampson HA, et al. Second symposium on the definition and management of anaphylaxis: summary report—Second National Institute of Allergy and Infectious Disease/Food Allergy and Anaphylaxis Network symposium. J Allergy Clin Imunol 2006; 117:391.

CRITERION 1

Acute onset of an illness (minutes to hours) involving the SKIN, MUCOSAL TISSUE, or both (hives, pruritis, swollen lips/tongue)

AND

RESPIRATORY COMPROMISE (dyspnea, wheeze, stridor, hypoxemia)

OR

REDUCED BP or associated sxs (i.e. syncope)

CRITERION 2
Helps identify patients without skin symptoms

TWO or MORE of the following, after exposure to a LIKELY ALLERGEN:

- Involvement of SKIN/MUCOSAL TISSUE
- RESPIRATORY COMPROMISE
- REDUCED BP or associated sxs
- Persistent GI sxs (crampy abdominal pain, vomiting)

CRITERION 3
Helps identify patients with isolated cardiovascular symptoms

REDUCED BP after exposure to a KNOWN allergen for that patient

In adults: systolic < 90mmHg, or

> 30% decrease from SBP baseline

In children: 1 mo - 1 year: < 70mmHg
1 - 10 yrs: < 70mmHg + (2x age)

Common Symptoms

- CUTANEOUS (90% of episodes) - flushing, itching, urticaria, angioedema
- RESPIRATORY (70%) - voice change, sensation of throat closure or choking, cough, wheeze, dyspnea, nasal discharge and/or congestion
- GASTROINTESTINAL (40%) - nausea, vomiting, diarrhea, crampy abdominal pain
- CARDIOVASCULAR (35%) - dizziness, tachycardia, hypotension, syncope

Differential Diagnosis

Common Disorders:
- Vasovagal syncope
- Acute generalized urticaria
- Acute angioedema
- Acute asthma exacerbation
- Vocal cord dysfunction
- Anxiety disorders
- Children: other causes of respiratory distress
- Adults: MI or stroke
- Other forms of shock

Differential Diagnosis

- Causes of flushing:
  - Medications (vancomycin, niacin)
  - Alcohol (EtOH - flushing may be exacerbated by meds)
  - Menopause
  - Tumors (carcinoid, VIPoma, pheochromocytoma)
- Rare disorders:
  - Mastocytosis, other clonal mast cell disorders
  - Certain leukemias - histamine excess
  - Hydatid cyst rupture
  - Capillary leak syndrome

Time Course

- Usually characterized by rapid onset, evolution, and resolution of symptoms in MINUTES TO HOURS
- May progress to cardiovascular or respiratory collapse within minutes

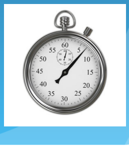

Biphasic Reactions

- Recurrence of symptoms after resolution WITHOUT ADDITIONAL EXPOSURE to the trigger
- Occur in 1-23% of episodes
- Occur within 8-10 hours after resolution of initial symptoms

Triggers of Anaphylaxis

IgE-mediated allergens:

- FOODS (peanut, tree nuts, shellfish, fish, milk, egg)
- Insect STINGS (esp. hymenoptera: wasps, bees, fire ants)
- LATEX
- MEDICATIONS (β-lactams)
- Biologic materials (monoclonal antibodies, vaccines)
- Food additives
- Inhalants (animal dander - rare)
- Seminal fluid
- Occupational allergens

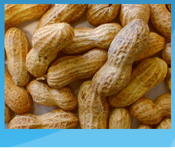

Triggers of Anaphylaxis

Non-IgE-mediated Immunologic Triggers

- IgG-dependent (rare - i.e. infliximab)
- Coagulation system activation

Idiopathic

- Mastocytosis or clonal mast cell disorders
- Autoimmunity

Non-Immunologic: direct activation of mast cells & basophils

- Physical (exercise, cold, heat, sunlight)
- Medications (opioids)
- Alcohol (EtOH)

Laboratory Diagnosis

- Clinical diagnosis is critical
- Diagnosis can be confirmed with the following:
- Plasma histamine
  - peaks 5-15 minutes after onset
  - declines to baseline by 60 minutes
- Serum or plasma tryptase
  - Must be obtained within 3 hours of onset
  - Not reliably elevated in food-induced anaphylaxis

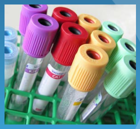

Immediate Management

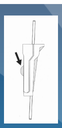

1) Remove the suspected inciting agent:
   i.e. STOP the medication infusion
   May apply tourniquet (injection/sting)
2) Call for help (RRT, 911)
3) AIRWAY - intubation may be urgent
4) Intramuscular injection of epinephrine (1:1000)
5) Supine, trendelenberg if tolerated
6) Volume resuscitation
7) Supplemental O2

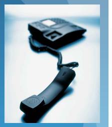

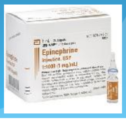

Epinephrine

- INTRAMUSCULAR injection into the mid-anterolateral thigh is the PREFERRED route (0.3 - 0.5 mg = 0.3 - 0.5 mL)
- IV epinephrine can be used when the patient doesn't respond to IM
- Epinephrine comes in several DILUTIONS - use caution:
  - INTRAMUSCULAR epinephrine: 1:1000
  - INTRAVENOUS epinephrine: 1:10,000
- Dose can be repeated in 5-15 minutes if symptoms do not resolve

Epinephrine

Epinephrine is the drug of choice, because it addresses the physiologic disturbances in anaphylaxis, better than any other drug:

α1 agonist - vasoconstriction, increased PVR, decreased mucosal edema (i.e. airway edema)

β1 agonist - inotropy and chronotropy

β2 agonist - bronchodilation, decreased release of inflammatory mediators from mast cells and basophils

Epinephrine – Adverse Effects

COMMON, TRANSIENT:
- anxiety
- restlessness
- dizziness
- headache
- palpitations
- pallor
- tremor

UNCOMMON, more often in overdose:
- ventricular arrhythmias
- angina
- myocardial infarction
- pulmonary edema
- sudden hypertension
- intracranial hemorrhage

Epinephrine: *Situations requiring caution*

There are NO absolute contraindications to the use of epinephrine in anaphylaxis

Subgroups of patients at theoretically higher risk w/ epi:
- Patients with cardiovascular disease
  - Anaphylaxis can unmask subclinical coronary disease, resulting in MI and/or arrhythmia – w/ or w/o epi
  - Heart is a target organ in anaphylaxis – mast cells throughout myocardium and coronary arteries
- MAOI/TCA – prolong epinephrine activity
- Preexisting conditions may place some at higher risk with epinephrine's actions: intracranial surgery, aortic aneurysm, uncontrolled hyperthyroid or HTN
- Concurrent use of stimulants (amphetamines, cocaine)

- Patients on β-blockers may be resistant to treatment with epinephrine
- Glucagon – inotropic and chronotropic effects not mediated through β receptors
  - Adults: 1-2 mg over 5 minutes, IV
  - Children: 20-30 mcg/kg up to 1 mg, IV

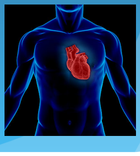

ADJUNCTIVE Therapies

- THERE IS NO SUBSTITUTE FOR EPINEPHRINE IN ANAPHYLAXIS!
- **H1 antihistamines** – treat Hives and Itching
  H1s do NOT treat - upper or lower airway obstruction
  gastrointestinal symptoms
  shock
- Second generation H1 (i.e. cetirizine): fewer side effects, less sedation, faster action, oral only
- First generation H1 (i.e. diphenhydramine): available IV

Adjunctive Therapies

- H2 blockers – minimal evidence to support use
- Bronchodilators – can be used for refractory airway obstruction after epinephrine ($\beta 2$), but do not relieve airway mucosal edema ($\alpha 1$)
- Glucocorticoids – onset takes hours, may prevent protracted or biphasic anaphylaxis, only 3-4 days, no taper needed
- Racemic epinephrine – helps treat stridor/upper airway obstruction

Care Upon Resolution

- Observation – minimally a few hours, prefer 8-10
- SAFE
  - Seek support – advise the life-threatening nature of event
    - symptoms of initial event may recur up to 72 hours
    - epinephrine, EMS, return to ER if recurrence
    - at risk for repeat anaphylaxis in the future
  - Allergen ID & Avoidance – through history, testing (CAP-RAST & skin prick)
  - Follow-up with allergist**
  - Epinephrine autoinjector for emergencies
- Anaphylaxis action plan

Outpatient Epinephrine

- Prescribe self-injectable epinephrine
  - Most commonly used device: EpiPen (0.3 mg)
  - EpiPen Jr. (0.15 mg) – patients < 30 kg
- Injection technique
- Carry at all times (often dispense 2)
- Indications for use

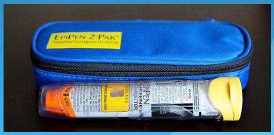

Appendix 2: Sample Pre-Test.

Please choose the one best answer for each question below:

1) A 24-year-old man with no significant medical history presents to the emergency department within 10 minutes after being stung by a bee. On examination, he appears to be short of breath, is wheezing diffusely, and reports the development of a diffuse urticarial rash within minutes of the sting. His blood pressure is 80/50 mm Hg and his face is swollen. Which of the following is recommended as an immediate first-line of treatment:
   a) Epinephrine (1:10,000) intravenously infused at a rate of 1-10 microgram/min
   b) Epinephrine (1:1,000) 0.3 mg intramuscularly
   c) Diphenhydramine 25 mg orally, intravenously, or intramuscularly
   d) Ranitidine 50 mg orally or intravenously
   e) Methylprednisolone 125 mg intravenously

2) The patient responds to your initial treatment. Which of the following laboratory tests, if available, would you check to help establish the diagnosis in this acute setting:
   a) Plasma histamine, if drawn after 2 hours of onset of symptoms
   b) Serum tryptase, if drawn after 6 hours of onset of symptoms
   c) Serum platelet activating factor, if drawn within 6 hours of onset of symptoms
   d) Serum platelet activating factor hydrolase, if drawn within 6 hours of onset of symptoms
   e) The diagnosis of acute anaphylaxis is primarily clinical; laboratory tests are not routinely needed for diagnosis

3) The patient continues to do well in the emergency department. After 1 hour of observation, the patient asks how much longer he needs to remain in the hospital. Based on the best available evidence-based recommendations, how should you respond to the patient:
   a) Biphasic anaphylaxis occurs in 1% - 23% of episodes of anaphylaxis, and symptoms may recur hours (most within 10 hrs) after apparent resolution of the initial phase. However, there are no reliable predictors of biphasic or protracted anaphylaxis available. We recommend continued observation in the emergency department for at least the next 2-8 hours.
   b) Biphasic anaphylaxis occurs in 1% - 23% of episodes of anaphylaxis, and symptoms may recur hours (most within 10 hrs) after apparent resolution of the initial phase. However, you responded well to the treatment, and can be discharged within the hour.
   c) Biphasic anaphylaxis occurs in 1% - 23% of episodes of anaphylaxis, and symptoms may recur hours (most within 10 hrs) after apparent resolution of the initial phase. We recommend admission for further inpatient serological testing and an allergy consultation.
   d) You responded well to the treatment, and can be discharged within the hour.

4) After a sufficient amount of observation, the patient is ready to be discharged home.

Which of the following is the next best step in this patient's management:

a) Provide a prescription for an Epinephrine autoinjector (e.g., EpiPen) along with a referral to an allergist for outpatient follow up

b) Provide a prescription for an Epinephrine autoinjector (e.g., EpiPen), demonstrate how to use the autoinjector correctly to the patient using a trainer, watch and critique the patient's technique with the trainer, along with a referral to an allergist for outpatient follow up.

c) Provide a prescription for Diphenhydramine 25 mg PO every 6 hours as needed and Ranitidine 50 mg PO daily as needed, along with a referral to an allergist for outpatient follow up.

d) Provide a prescription for a short course of glucocorticoid taper (e.g., Medrol Dose Pack), along with a referral to an allergist for outpatient follow up.

5) A 90-year-old female nursing home resident with past medical history significant for dementia, depression, hypertension, and known penicillin allergy was found to be febrile with a positive urinalysis. She has been admitted for the treatment of presumed urosepsis. On the floor, the nurse administers her first dose of Ceftriaxone 1 gm IV, and a rapid response is called within minutes as the patient is noted to have difficulty breathing, pulse of 120/min, BP of 90/50 mm Hg, and oxygen saturation of 90% on 2 L nasal cannula. You are the leader of the rapid response team. Which of the following is the next best step in this patient's management:

a) Ensure that the Ceftriaxone infusion has been discontinued, administer intramuscular epinephrine, and ensure that ABC (airway, breathing, circulation) are secured

b) Ensure that ABC (airway, breathing, circulation) are secured, administer intramuscular epinephrine, and reposition the patient in a recumbent position while elevating her lower extremities

c) Ensure that ABC (airway, breathing, circulation) are secured, administer intravenous methylprednisolone, and administer intramuscular epinephrine if patient does not clinically improve after the administration of corticosteroid

d) Ensure that ABC (airway, breathing, circulation) are secured, administer intravenous diphenhydramine or ranitidine, and administer intramuscular epinephrine if patient does not clinically improve after the administration of antihistamine

6) During the rapid response, you request that epinephrine be administered to the patient. One of the team members prepares the epinephrine as directed, and asks how the medication should be administered. Based on the best available evidence-based recommendations, how should the epinephrine be administered?

a) intramuscularly in the deltoid

b) subcutaneously in the abdomen

c) intramuscularly in the upper, outer quadrant of the buttock

d) subcutaneously in the lateral thigh

e) intramuscularly in the lateral thigh

7) When, if ever, should a second or subsequent dose of epinephrine be administered in

a patient experiencing anaphylaxis:
a) After 5-15 minutes if the patient does not improve
b) After 30 minutes if the patient does not improve
c) After 60 minutes if the patients does not improve
d) A second dose should never be administered

8) As the rapid response ensues, you review the patient's medication list. All of the following medications may affect the efficacy of epinephrine in the treatment of anaphylaxis except:
a) Beta blockers such as Metoprolol
b) ACE inhibitors such as Lisinopril
c) Angiotensin receptor inhibitors such as Losartan
d) Diuretics such as Hydrochlorothiazide
e) Tricyclic antidepressants such as Amitriptyline

9) A patient taking a Beta Blocker may be resistant to treatment with Epinephrine. Which of the following is the most appropriate intervention in such a situation:
a) Glucagon 20-30 mcg/kg, up to 1 mcg intravenously
b) Glucagon 1-2 mg intravenously, over 5 min
c) Glucagon 20-30 mcg/kg, up to 1 mcg intramuscularly
d) Glucagon 1-2 mg intramuscularly

10) Although the patient responds to the treatment and becomes hemodynamically stable, she remains febrile and is transferred to the medical intensive care unit for closer monitoring. The preliminary microbiological reports from the patient's urine and blood cultures are positive for gram-negative cocci, but the final identification and antibiotic susceptibilities will not be ready for at least another 12 hours. The ICU attending instructs you to continue antibiotic coverage based on the preliminary findings. What of the following is the best next course of action:
a) Request emergent Infectious Disease and Allergy consultations. Consider use of an alternative cephalosporin such as Cefepime in the meantime.
b) Request emergent Infectious Disease and Allergy consultations. Consider use of a beta-lactam antibiotic such as Ampicillin in the meantime.
c) Request emergent Infectious Disease and Allergy consultations. Consider use of a monobactam antibiotic such as Aztreonam in the meantime.
d) Request emergent Infectious Disease and Allergy consultations. Consider use of a combination antibiotic such as Piperacillin/Tazobactam in the meantime.

Appendix 3: Sample Post-Test and Follow-Up Test.

Please choose the one best answer for each of the questions below:

1. A patient taking a Beta Blocker may be resistant to treatment with Epinephrine. Which of the following is the most appropriate intervention in such a situation:
a. Glucagon 20-30 mcg/kg, up to 1 mcg intravenously
b. Glucagon 1-2 mg intravenously, over 5 min
c. Glucagon 20-30 mcg/kg, up to 1 mcg intramuscularly
d. Glucagon 1-2 mg intramuscularly

2. A 97-year-old male nursing home resident with past medical history significant for dementia, depression, coronary artery disease s/p percutaneous coronary intervention, and known penicillin allergy was found to be febrile with a positive urinalysis. He has been admitted for the treatment of presumed urosepsis. On the floor, the nurse administers the first dose of Ceftriaxone 1 gm IV, and a rapid response is called within minutes as the patient is noted to be dyspneic, with pulse of 120/min, BP of 90/50 mm Hg, and oxygen saturation of 89% on 4 L nasal cannula. You are the leader of the rapid response team. Which of the following is the next best step in this patient's management:
a. Ensure that the Ceftriaxone infusion has been discontinued, administer intramuscular epinephrine, and ensure that ABC (airway, breathing, circulation) are secured
b. Ensure that ABC (airway, breathing, circulation) are secured, administer intramuscular epinephrine, and reposition the patient in a recumbent position while elevating her lower extremities
c. Ensure that ABC (airway, breathing, circulation) are secured, administer intravenous methylprednisolone, and administer intramuscular epinephrine if patient does not clinically improve after the administration of corticosteroid
d. Ensure that ABC (airway, breathing, circulation) are secured, administer intravenous diphenhydramine or ranitidine, and administer intramuscular epinephrine if patient does not clinically improve after the administration of antihistamine

3. You review the patient's medication list. All of the following medications may affect the efficacy of epinephrine in the treatment of anaphylaxis except:
a. Tricyclic antidepressants such as Amitriptyline
b. ACE inhibitors such as Lisinopril
c. Angiotensin receptor inhibitors such as Losartan
d. Diuretics such as Hydrochlorothiazide
e. Beta blockers such as Metoprolol

4. During the rapid response, you request that epinephrine be administered to the patient. The nurse asks how the medication should be administered. Based on the best available evidence-based recommendations, how should the epinephrine be administered?

a. intramuscularly in the deltoid
b. intramuscularly in the lateral thigh
c. intramuscularly in the upper, outer quadrant of the buttock
d. subcutaneously in the lateral thigh
e. subcutaneously in the abdomen

5. When, if ever, should a second or subsequent dose of epinephrine be administered in a patient experiencing anaphylaxis:

a. After 5 minutes if the patient does not improve
b. After 15 minutes if the patient does not improve
c. After 30 minutes if the patients does not improve
d. A second dose should never be administered

6. Although the patient responds to the treatment and becomes hemodynamically stable, she remains febrile and is transferred to the medical intensive care unit for closer monitoring. The preliminary microbiological reports from the patient's urine and blood cultures are positive for gram-negative cocci, but the final identification and antibiotic susceptibilities will not be ready for at least another 12 hours. The ICU attending instructs you to continue antibiotic coverage based on the preliminary findings. What of the following is the best next course of action:

a. Request emergent Infectious Disease and Allergy consultations. Consider use of an alternative cephalosporin such as Cefepime in the meantime.
b. Request emergent Infectious Disease and Allergy consultations. Consider use of a beta-lactam antibiotic such as Ampicillin in the meantime.
c. Request emergent Infectious Disease and Allergy consultations. Consider use of a monobactam antibiotic such as Aztreonam in the meantime.
d. Request emergent Infectious Disease and Allergy consultations. Consider use of a combination antibiotic such as Piperacillin/Tazobactam in the meantime.

7. A 24-year-old man with mild, intermittent asthma presents to the emergency department within 10 minutes after being stung by a wasp. On examination, he appears to be short of breath, is wheezing diffusely, and reports the development of a diffuse urticarial rash within minutes of the sting. His blood pressure is 80/50 mm Hg and his face is swollen. Which of the following is recommended as an immediate first-line of treatment:

a. Methylprednisolone 125 mg intravenously
b. Epinephrine (1:1,000) 0.3 mg intramuscularly
c. Epinephrine (1:10,000) intravenously infused at a rate of 1-10 microgram/min

d. Ranitidine 50 mg orally or intravenously
e. Diphenhydramine 25 mg orally, intravenously, or intramuscularly

8. The patient responds to your initial treatment. Which of the following laboratory tests, if available, would you check to help establish the diagnosis in this acute setting:
a. The diagnosis of acute anaphylaxis is primarily clinical; laboratory tests are not routinely needed for diagnosis
b. Serum tryptase, if drawn after 6 hours of onset of symptoms
c. Serum platelet activating factor, if drawn within 6 hours of onset of symptoms
d. Serum platelet activating factor hydrolase, if drawn within 6 hours of onset of symptoms
e. Plasma histamine, if drawn after 2 hours of onset of symptoms

9. The patient continues to do well in the emergency department. After 1 hour of observation, the patient asks how much longer he needs to remain in the hospital. Based on the best available evidence-based recommendations, how should you respond to the patient:
a. You responded well to the treatment, and can be discharged within the hour.
b. Biphasic anaphylaxis occurs in 1% - 23% of episodes of anaphylaxis, and symptoms may recur hours (most within 10 hrs) after apparent resolution of the initial phase. However, you responded well to the treatment, and can be discharged within the hour.
c. Biphasic anaphylaxis occurs in 1% - 23% of episodes of anaphylaxis, and symptoms may recur hours (most within 10 hrs) after apparent resolution of the initial phase. We recommend admission for further inpatient serological testing and an allergy consultation.
d. Biphasic anaphylaxis occurs in 1% - 23% of episodes of anaphylaxis, and symptoms may recur hours (most within 10 hrs) after apparent resolution of the initial phase. However, there are no reliable predictors of biphasic or protracted anaphylaxis available. We recommend continued observation in the emergency department for at least the next 2-8 hours.

10. After a sufficient amount of observation, the patient is ready to be discharged home. Which of the following is the next best step in this patient's management:
a. Provide a prescription for a short course of glucocorticoid taper (e.g., Medrol Dose Pack), along with a referral to an allergist for outpatient follow up.
b. Provide a prescription for an Epinephrine autoinjector (e.g., EpiPen) along with a referral to an allergist for outpatient follow up.
c. Provide a prescription for an Epinephrine autoinjector (e.g., EpiPen), demonstrate how to use the autoinjector correctly to the patient using a trainer,

watch and critique the patient's technique with the trainer, along with a referral to an allergist for outpatient follow up.

d. Provide a prescription for Diphenhydramine 25 mg PO every 6 hours as needed and Ranitidine 50 mg PO daily as needed, along with a referral to an allergist for outpatient follow up.

Appendix 4: Template for Pre-test questionnaire.

Please choose the one best answer for each of the questions below:

1. I am	MALE FEMALE
2. I am currently a	PGY-1 PGY-2 PGY-3
3. I graduated from medical school in the USA	TRUE FALSE
4. I have diagnosed anaphylaxis in the past	TRUE FALSE
5. I have been involved in managing anaphylaxis in the past<br>If TRUE, please specify:	TRUE FALSE<br>Floors Intensive Care Emergency Dept<br>Other (specify)
6. I have used an epinephrine autoinjector (e.g., EpiPen, Adrenaclick, Twinject) in the past	TRUE FALSE
7. I have demonstrated use of an epinephrine autoinjector (e.g., EpiPen, Adrenaclick, Twinject) to another individual in the past	TRUE FALSE
8. I have referred an anaphylaxis patient to an allergist for further evaluation in the past	TRUE FALSE
9. I feel confident in my ability to diagnose anaphylaxis	TRUE FALSE
10. I feel confident in my ability to manage anaphylaxis	TRUE FALSE

Appendix 5: Template for Post-test questionnaire and Follow up questionnaire

Please choose the one best answer for each of the questions below:

1. I am	MALE FEMALE
2. I am currently a	PGY-1 PGY-2 PGY-3
3. I graduated from medical school in the USA	TRUE FALSE
4. I have diagnosed anaphylaxis in the past	TRUE FALSE
5. I have been involved in managing anaphylaxis in the past	TRUE FALSE
6. I have used an epinephrine autoinjector (e.g., EpiPen, Adrenaclick, Twinject) in the past	TRUE FALSE
7. I have demonstrated use of an epinephrine autoinjector (e.g., EpiPen, Adrenaclick, Twinject) to another individual in the past	TRUE FALSE
8. I have referred an anaphylaxis patient to an allergist for further evaluation in the past	TRUE FALSE
9. I feel confident in my ability to diagnose anaphylaxis	TRUE FALSE
10. I feel confident in my ability to manage anaphylaxis	TRUE FALSE
11. I participated in a survey on anaphylaxis diagnosis and management earlier this academic year	TRUE FALSE
12. I participated in an educational session on anaphylaxis diagnosis and management presented by the Division of Allergy & Immunology earlier this academic year	TRUE FALSE

Please continue to choose the one best answer for the following questions below *only* if you answered "TRUE" to question 12 above.

13. I have diagnosed anaphylaxis since I completed the educational session	TRUE FALSE
14. I have been involved in managing anaphylaxis since I completed the educational session	TRUE FALSE
15. I have used an epinephrine autoinjector (e.g., EpiPen) since I completed the educational session	TRUE FALSE
16. I have demonstrated how to use an epinephrine autoinjector (e.g., EpiPen) since I completed the educational session	TRUE FALSE
17. I have referred an anaphylaxis patient to an allergist for further evaluation since I completed the educational session	TRUE FALSE
18. I feel more confident in my ability to diagnose anaphylaxis since I completed the educational session	TRUE FALSE
19. I feel more confident in my ability to manage anaphylaxis since I completed the educational session	TRUE FALSE
